# Electrical Conductivity of Subsurface Ocean Analogue Solutions from Molecular Dynamics Simulations

**DOI:** 10.1021/acsearthspacechem.3c00345

**Published:** 2024-06-08

**Authors:** Catherine A. Psarakis, Timothy Tizhe Fidelis, Keith B. Chin, Baptiste Journaux, Abby Kavner, Pranab Sarker, Marshall J. Styczinski, Steven D. Vance, Tao Wei

**Affiliations:** † University of California, Los Angeles, Los Angeles, California 90095, United States; ‡ Jet Propulsion Laboratory, California Institute of Technology, Pasadena, California 91011, United States; § Howard University, Washington, District of Columbia 20059, United States; ∥ University of Washington, Seattle, Seattle, Washington 98195, United States; ⊥ University of South Carolina, Columbia, South Carolina 29208, United States; # Blue Marble Space Institute of Science, Seattle, Washington 98104, United States

**Keywords:** molecular mechanics, dynamics, conductivity, computational chemistry, applications of chemistry, solutions, solvents, physical properties

## Abstract

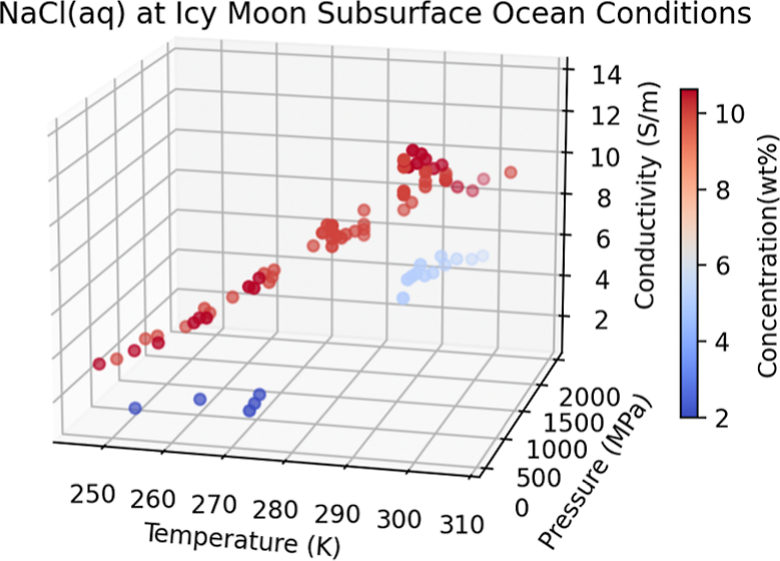

Investigating the habitability of ocean worlds is a priority of current and future NASA missions. The *Europa Clipper* mission will conduct approximately 50 flybys of Jupiter’s moon Europa, returning a detailed portrait of its interior from the synthesis of data from its instrument suite. The magnetometer on board has the capability of decoupling Europa’s induced magnetic field to high precision, and when these data are inverted, the electrical conductivity profile from the electrically conducting subsurface salty ocean may be constrained. To optimize the interpretation of magnetic induction data near ocean worlds and constrain salinity from electrical conductivity, accurate laboratory electrical conductivity data are needed under the conditions expected in their subsurface oceans. At the high-pressure, low-temperature (HPLT) conditions of icy worlds, comprehensive conductivity data sets are sparse or absent from either laboratory data or simulations. We conducted molecular dynamics simulations of candidate ocean compositions of aqueous NaCl under HPLT conditions at multiple concentrations. Our results predict electrical conductivity as a function of temperature, pressure, and composition, showing a decrease in conductivity as the pressure increases deeper into the interior of an icy moon. These data can guide laboratory experiments at conditions relevant to icy moons and can be used in tandem to forward-model the magnetic induction signals at ocean worlds and compare with future spacecraft data. We discuss implications for the *Europa Clipper* mission.

## 1 Plain Language Summary

Oceans in the solar system are of great interest due to their potential for past or present life. If the electrical conductivity profile can be inferred from magnetic data, then a range of possible compositions of the ocean can be determined from the relationship between conductivity and concentration of dissolved solids, which we find from laboratory experiments. New magnetometers, such as the one onboard the planned *Europa Clipper* mission, will have much greater precision in measuring the induced field of icy moons than is available with existing data and will require a higher-precision data set of electrical conductivity at the high-pressure and low-temperature conditions present in ocean world interiors to make the most of the data.

Here, we describe simulations of NaCl dissolved in water under conditions relevant to icy moons in the solar system. Our results predict electrical conductivity as a function of temperature, pressure, and composition. The new data set shows a decrease in conductivity with increasing pressure deeper into the interior of an icy moon. The new results can guide future laboratory experiments that can be used to interpret spacecraft data. We discuss implications for the upcoming *Europa Clipper* mission.

## 2 Introduction

The subsurface oceans of icy moons are the most promising places to search for life beyond Earth.^1^ They also serve as analogues to watery exoplanets. There is mounting evidence that liquid water is common inside icy bodies throughout the outer solar system, even as far from the Sun as Pluto.^2^

Magnetic measurements have proven vital for constraining the properties of oceans in icy moons.^3,4^ Specifically, oscillations in the magnetic field applied by the parent planet generate electric currents within the electrically conducting subsurface oceans, creating a secondary induced magnetic field that can be measured at orbital distances by spacecraft. Because the physicochemical properties of subsurface oceans affect their electrical conductivity and thus the induced magnetic fields measured by spacecraft, *magnetic sounding* investigations can be used in conjunction with laboratory measurements of electrical conductivity versus pressure, temperature, and composition to provide bounds on salinity.^5,6^

Previous studies of Europa have used magnetic sounding data from the *Galileo* mission to constrain the range of possible salinity and electrical conductivity of its subsurface ocean from the induced magnetic field. Most notably, Hand and Chyba^4^ used induced field limits derived by Zimmer et al.^7^ and Schilling et al.^8^ to put upper and lower bounds on Europa’s ocean conductivity. Available laboratory measurements of electrical conductivity were used to infer possible ocean salinities. However, the limited experimental data available required extrapolation to the relevant conditions. Upcoming missions NASA’s *Europa Clipper*(9) and ESA’s *JUICE* will measure the induced magnetic field with better sampling of the frequency-dependent response,^10^ motivating improvements in precision of laboratory measurements and modeling efforts that can utilize the enhanced data.

The Europa Clipper Magnetometer (ECM) will sample the magnetic field near Europa with good coverage of Europa's orbital mean anomaly and Jupiter's magnetic phase (system III longitude), enabling retrieval of the induction response (phase and amplitude) for both the 11.2 h synodic period and for the longer (85.2 h) orbital-period oscillation.^11^ To maximize the science return from this magnetic field investigation, the electrical conductivity of the relevant aqueous solutions must be precisely constrained. The electrical conductivity data set used by Hand and Chyba^4^ consisted of measurements of candidate ocean solutions of NaCl and MgSO_4_ dissolved in liquid water from previous studies at ambient pressure (0.1 MPa) and at 0 °C or 25 °C. This study was crucial in establishing a means to infer ocean salinity from the induced field of a moon. However, appropriate laboratory measurements for NaCl and MgSO_4_ solutions were not available at the predicted pressures and temperatures in the ocean, so this study did not take advantage of the link between ice thickness and ocean temperature that affects the magnetic induction response.^5^

Currently available experimental data for salt solutions are inadequate for the high-pressure and low-temperature conditions of icy moon subsurface oceans. For one of the most likely candidate salts, NaCl, the majority of published conductivity data at high pressure were obtained at ambient temperature (around 298 K), lower salinity (<5 wt %), and at only a few selected pressures (1000 and 2000 MPa by Guo and Keppler;^12^ 0–99 MPa by Sinmyo and Keppler^13^). One exception—a study by Adams and Hall^14^—measured conductivity at up to 400 MPa for a higher salinity of 10 wt % NaCl. Pan et al.^15^ measured conductivity for NaCl solutions in a high-pressure, low-temperature range (212–1713 MPa and 233–295 K) above 200 MPa that is relevant to the larger ocean worlds Ganymede, Callisto, and Titan.^16^ However, many of the measurements by Pan et al.^15^ were at temperatures below the melting points of the fluids, and therefore, in a solid–liquid two-phase system, making the interpretation of those data inconclusive.

Pan et al.^15^ supplemented their recent investigation of conductivity at high pressure with a series of molecular dynamics (MD) simulations designed to infer the ion diffusivity and associated electrical conductivity at conditions relevant to Europa’s ocean (0–200 MPa). MD simulations have been increasingly used to calculate the conductivity of aqueous solutions in recent years, as they offer additional insights into the properties of ionic liquids at the molecular scale, and are not subject to the same experimental limitations as laboratory investigations.^17^ These simulations predict diffusion coefficients of the relevant ions in a solution, which may be used to derive electrical conductivity via the Nernst–Einstein relation.^18^

**Figure 1:**
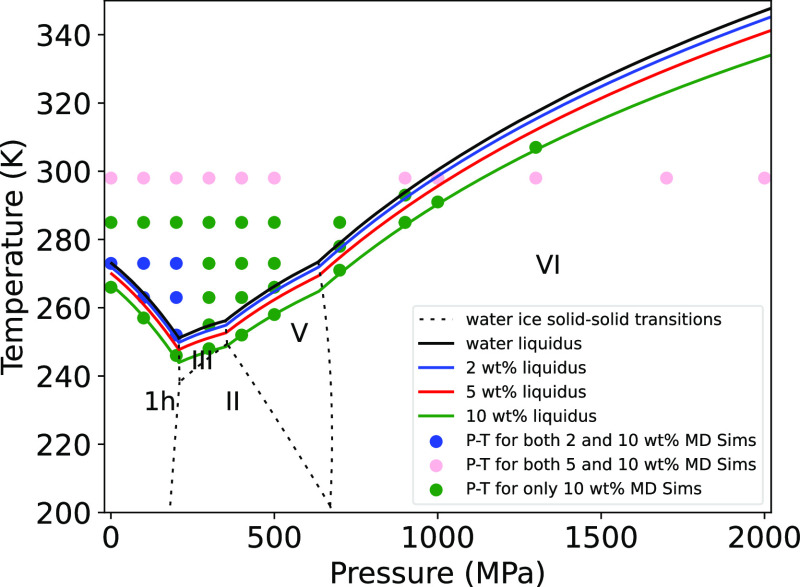
Catalog of conditions for our MD simulations, applying to both TIP4P and SPC/E water models, plotted with the phase diagram of the NaCl–H_2_O system as evaluated by *SeaFreeze*.^19,20^

Here, we report the results of new MD simulations of aqueous NaCl at 0–2000 MPa and 246–307 K, above the freezing point of the NaCl– H_2_O mixture. The freezing point was calculated for each studied solution using ambient pressure corrections as a function of concentration using *SeaFreeze*.^19,20^ Our simulations overlap selected conditions from Pan et al.^15^ (see Figure 1) while filling the liquid area above the melting curve and extending beyond 1000 MPa to provide comprehensive pressure and temperature coverage in much of the liquid stability range for high concentrations. These results allow us to assess the current utility of MD simulations as a companion to laboratory experiments intended to support ocean world magnetic induction investigations.

## 3 Methods

MD simulations were performed for NaCl in H_2_O using periodic boundary conditions and a duration of 300 ns using GROMACS software version 2019.6.^21,22^ Two different water models—SPC/E and TIP4P—were employed, and 2, 5, and 10 wt % NaCl (aq) ions were modeled as Lennard-Jones particles. The ion pairs and total particles for each concentration are listed in the Supporting Information (Table S5). Force field parameters for each pair of atoms were defined using the CHARMM36 force field.^23^ First, MD runs were performed in an isochoric–isothermal canonical ensemble (*NVT*). Previous MD studies^24,25^ have shown this approach to equilibrate the system efficiently and repeatably when run before performing the subsequent simulations in an isobaric–isothermal ensemble (*NPT*). We used the final configuration at the target temperature from the *NVT* run as the input structure for a 300 ns *NPT* run at the desired pressure. This *NVT*-then-*NPT* simulation strategy has been shown to more accurately estimate the particle motion at the given conditions than either ensemble alone.^24−26^ The diffusion coefficient for ions is linearly proportional to the mean-squared displacement versus time^18^
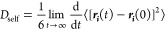
1The electrical conductivity σ, in S/m, was computed from the diffusion coefficients of both cations (*D*_+_) and anions (*D*_–_) using the Nernst–Einstein relation^18^
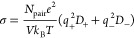
2with *N*_pair_ the number of ion pairs in the simulation box, *e* the electron charge, *V* the output simulation box volume, *k*_B_ the Boltzmann constant, *T* the temperature, and *q*_±_ the effective charge state for each type of ion.

## 4 Results

**Figure 2:**
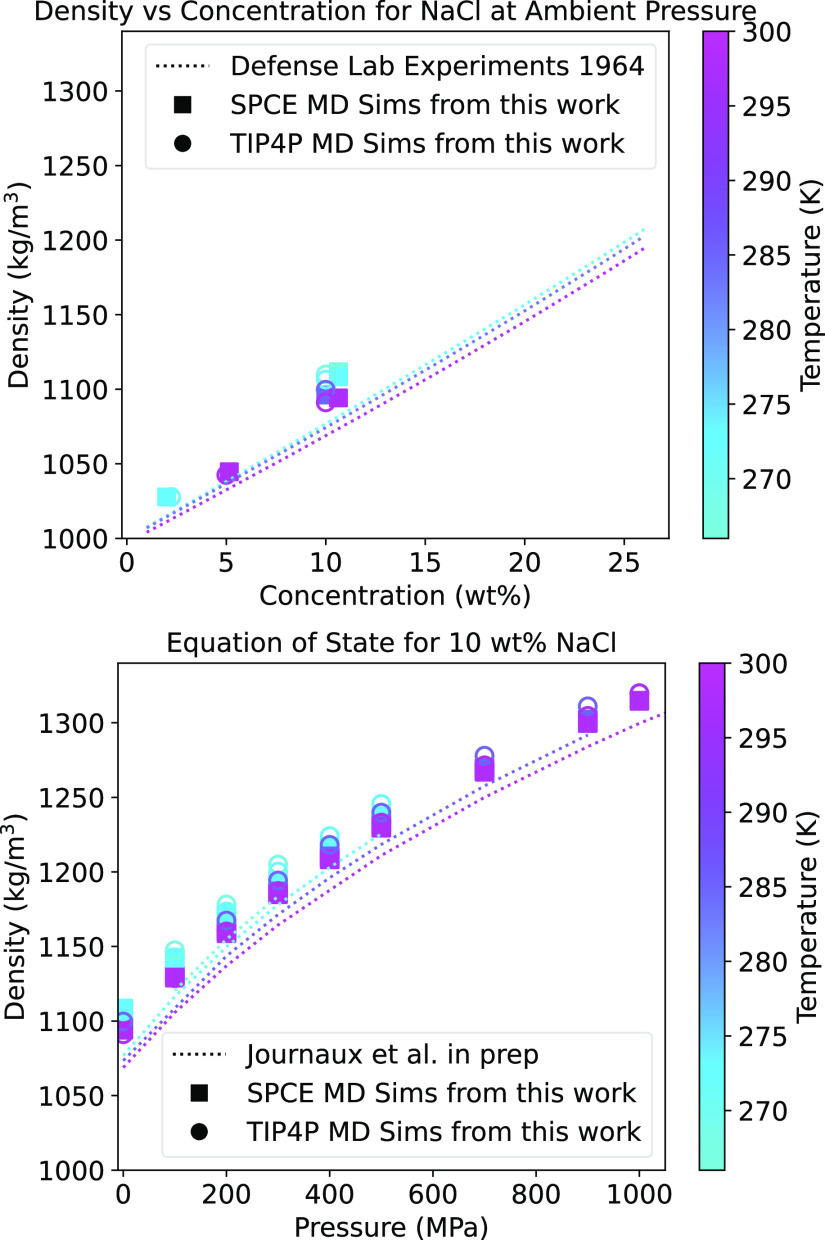
Top: density of aqueous NaCl vs concentration from our MD simulations compared with available data.^27^ Bottom: density of aqueous NaCl vs pressure from our MD simulations, compared with densities computed using *SeaFreeze*,^19,20^ which closely match the densities derived by Mantegazzi et al.^28^ in that pressure and temperature range. Although the MD densities are slightly larger than the accepted values, the slope and curvature of density versus pressure are in agreement. The temperature dependence also agrees with accepted values.

**Figure 3:**
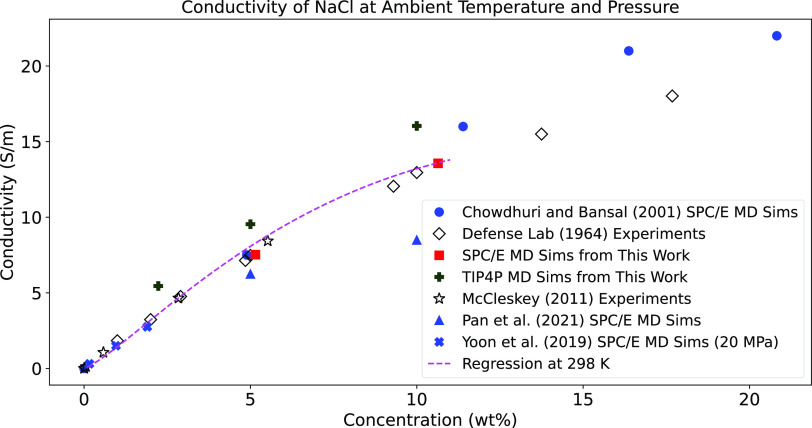
Conductivity vs concentration (in wt % NaCl) for the MD simulations of this study and prior experimental and simulation literature. Regression curves with eq 3 using SPC/E data are shown at 298 K. Note that the regressions are fit to the entire SPC/E data set, not just the two SPC/E data points at ambient temperature shown here. The laboratory experiments from McCleskey^31^ and Kraichman^32^ show better agreement with our MD simulations that used the SPC/E water model. The low-concentration simulations by Yoon et al.^33^ and Chowdhury and Bansal^34^ (who also use SPC/E) align with our SPC/E results.

**Figure 4:**
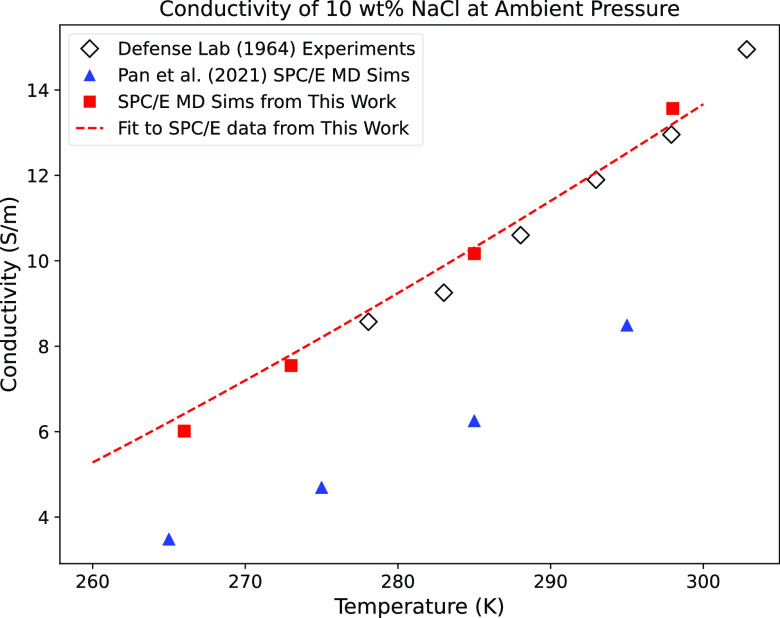
Conductivity vs temperature for our MD simulations compared with experimental data from the literature. A regression curve from the eq 3 fit using SPC/E data is shown. All depicted data are at ambient pressure and a concentration of 10 wt % NaCl. A subset of data for the SPC/E model had concentrations of 10.64 wt % for historical comparisons; these data were scaled to 10 wt % based on an assumption of a linear relationship between conductivity and concentration in this range.

The primary outputs of the simulations are bulk density and conductivity, and a subset of our outputs (e.g., those at ambient conditions) can be used to benchmark the simulations against prior measurements (Figures 2–4). Although the results of the SPC/E model reproduce laboratory measurements of conductivity as a function of temperature and concentration at ambient pressure (Figures 3 and 4), both models overestimate expected densities: SPC/E by ∼1.8% and TIP4P by ∼2.5% as compared to equation-of-state values from Journaux et al. (in prep), as shown in Figure 2. These differences in density may derive from the varying molecular bond angles and properties of the SPC/E and TIP4P water potential models, as well as the ability for each model to reproduce ion pairing in solution.^29,30^ The TIP4P and SPC/E model data have slopes of density as a function of *T*, *P*, and *m* that are nearly identical to each other (Table S1) and that are similar to those of the equation of state (Figure 2). Therefore, we interpret our results as indicating that both models capture important physical behavior of the NaCl/H_2_O system under the studied conditions.

Our data show that the conductivity values at each concentration inferred from the TIP4P model are higher than those of the SPC/E model. SPC/E conductivity values better match the values of previous MD simulations and laboratory experiments available in the literature.^33,34^ This may stem from the SPC/E model being designed to reproduce transport properties like conductivity.^29^ Additionally, the conductivities of SPC/E and TIP4P give significantly different dependences on concentration, with TIP4P having a stronger variation in conductivity vs concentration (Figure 3). The TIP4P model also shows a stronger temperature dependence of the conductivity (Table S1). As with the concentration dependence, the SPC/E temperature dependence better matches prior experimental literature (Figure 4).

Since the SPC/E conductivity values and dependence as a function of concentration and temperature match the previous literature better than those from the TIP4P model, we used the SPC/E data to construct a regression for conductivity as a function of *T* in K, *P* in MPa, and *m* in wt % NaCl. Our regression equation is of Arrhenius form, modified from that of Zhang et al.^35^ to include a pressure term
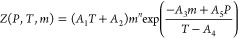
3where *A*_1_–*A*_5_ and *n* are fit parameters. The best-fit parameters from our SPC/E simulations are listed in Table 1. Fit parameters for TIP4P simulations, and all simulations combined, are provided in the Supporting Information (Tables S1 and S2). Uncertainty of evaluated conductivities using eq 3 is propagated via^36^
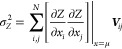
4where σ_*Z*_ is the uncertainty in *Z* and *i* and *j* iterate over the fit parameters *x* = {*x*_*i*_} = {*A*_1_, *A*_2_, ... } with mean values μ. Covariance matrices *V*_*ij*_ for the various fits are included in the Supporting Information.

**Table 1:** Best-Fit Parameters for Eq 3 Using Our SPC/E Simulation Runs at All Temperatures, Pressures, and NaCl Concentrations^a^

fit parameter	best-fit value
*A*_1_	0.0220(45)
*A*_2_	–5.0(11)
*A*_3_	18(11)
*A*_4_	62(98)
*A*_5_	–0.050(21)
*n*	1.26(18)

a Fits to other combinations of our model runs are in the Supporting Information.

The fit equation we adapted from Zhang et al.^35^ to reach eq 3 uses concentration units of molal (mol NaCl/kg H_2_O), which does not map linearly to the units we use, wt % NaCl (% NaCl by mass of solution). We also fit our conductivity data from the MD simulations to the same equation with concentrations expressed in molal. The results are in Table S2 and demonstrate goodness-of-fit parameters nearly identical to those found with concentrations in wt %.

A plot of the fit using the regression from Table 1 with our SPC/E data for conductivity as a function of concentration at multiple temperatures is shown in Figure 3 along with our SPC/E and TIP4P results and data from previous experimental studies. Since our SPC/E data only extend to 10 wt %, we also performed regression analysis using our SPC/E data combined with data from Kraichman^32^ to extend to higher concentrations. A similar comparison for our regression using SPC/E data is shown in Figure 4 as a function of the temperature. We note that the Pan et al.^15^ SPC/E MD simulations show much lower conductivity values as a function of temperature compared to our simulations, which correspond to the published experiments of Kraichman.^32^ We do not have enough information from this work to determine why, but we observe that their simulations use the *NPT* method followed by the *NVT* method (the inverse of our procedure) and a shorter equilibration time.^15^ Pan et al.^15^ did not make experimental measurements at 1 bar, and we cannot compare their simulations with experiments in this range.

**Figure 5:**
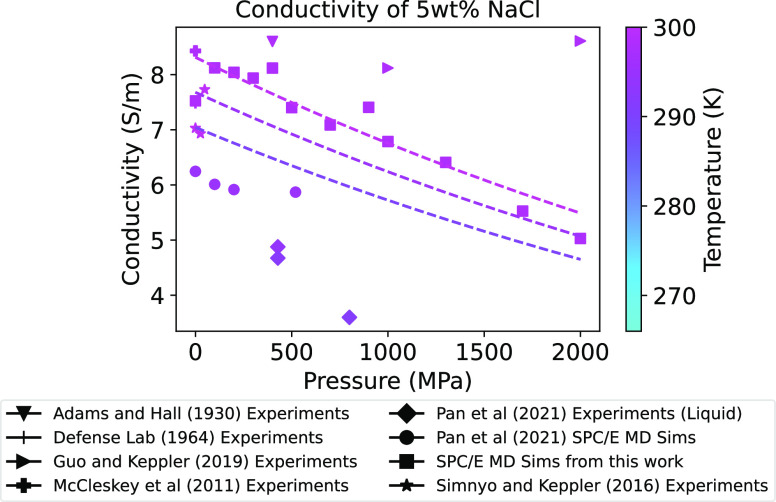
Conductivity of 5 wt % NaCl vs pressure. Our MD simulation results using the SPC/E water model are compared with published measurements and MD simulations. Regression curves fit to eq 3 using SPC/E data are shown. Note that the Pan et al.^15^ experimental data shown are a subset of conductivity measurements, including only those in the liquid-only phase. MD simulations by Pan et al.^15^ are also shown for comparison. Exact concentration, temperature, and pressure conditions for these data are shown in Table S6.

Because the SPC/E conductivity values better match published data in their dependence on concentration and temperature, we also used our SPC/E results to investigate the less understood pressure dependence. As shown in Figure 5, conductivity decreases with pressure for our SPC/E MD simulations, as well as for the experimental data from Adams and Hall^14^ and Pan et al.^15^ and MD simulations from Pan et al.^15^ It is important to note that the data in Figure 5 are not exactly at the same temperature and concentration. As shown in Figures 3 and 4, conductivity is highly dependent on concentration and temperature, which may contribute to the spread of pressure-dependent data. The exact conditions for these data are listed in Table S6. Our MD simulations provide a model for the pressure dependence on conductivity in addition to the dependence on temperature and concentration and address the gap in the insufficient data at high pressure. This is the best model at present to represent conductivity under ocean world conditions. A priority for future experiments is to confirm the pressure dependence exhibited by the MD simulation fit and improve the precision of the fit for use in geophysical forward modeling.

## 5 Conclusions and Implications for the *Europa Clipper* Magnetic Investigation

In the context of the planned magnetic investigations of Europa, we assess the needed precision of electrical conductivity data equivalent to the anticipated measurement uncertainty of the induction response. Assuming a measurement sensitivity consistent with that expected for the ECM^11^ of Δ*B* = 1.5 nT, we compute the noise-equivalent increment of conductivity for Europa using the available frameworks *PlanetProfile*(37,38) and *MoonMag*.^39^ This quantity establishes the precision required of laboratory and/or MD simulation data to not be a dominant source of uncertainty in the magnetic sounding investigation. The calculation propagates uncertainty for a single dependent variable: Δ*X*_B_ = Δ*B*∂*X*/∂*B*_max_. For the example of Europa, we use a magnetic excitation at a synodic period with Jupiter of *B*_syn_ = 209.78 nT.^5^ The maximum of the periodic induced field is the predicted amplitude of the induction response at the excitation period times the excitation amplitude: *A*_syn_*B*_syn_.

**Figure 6:**
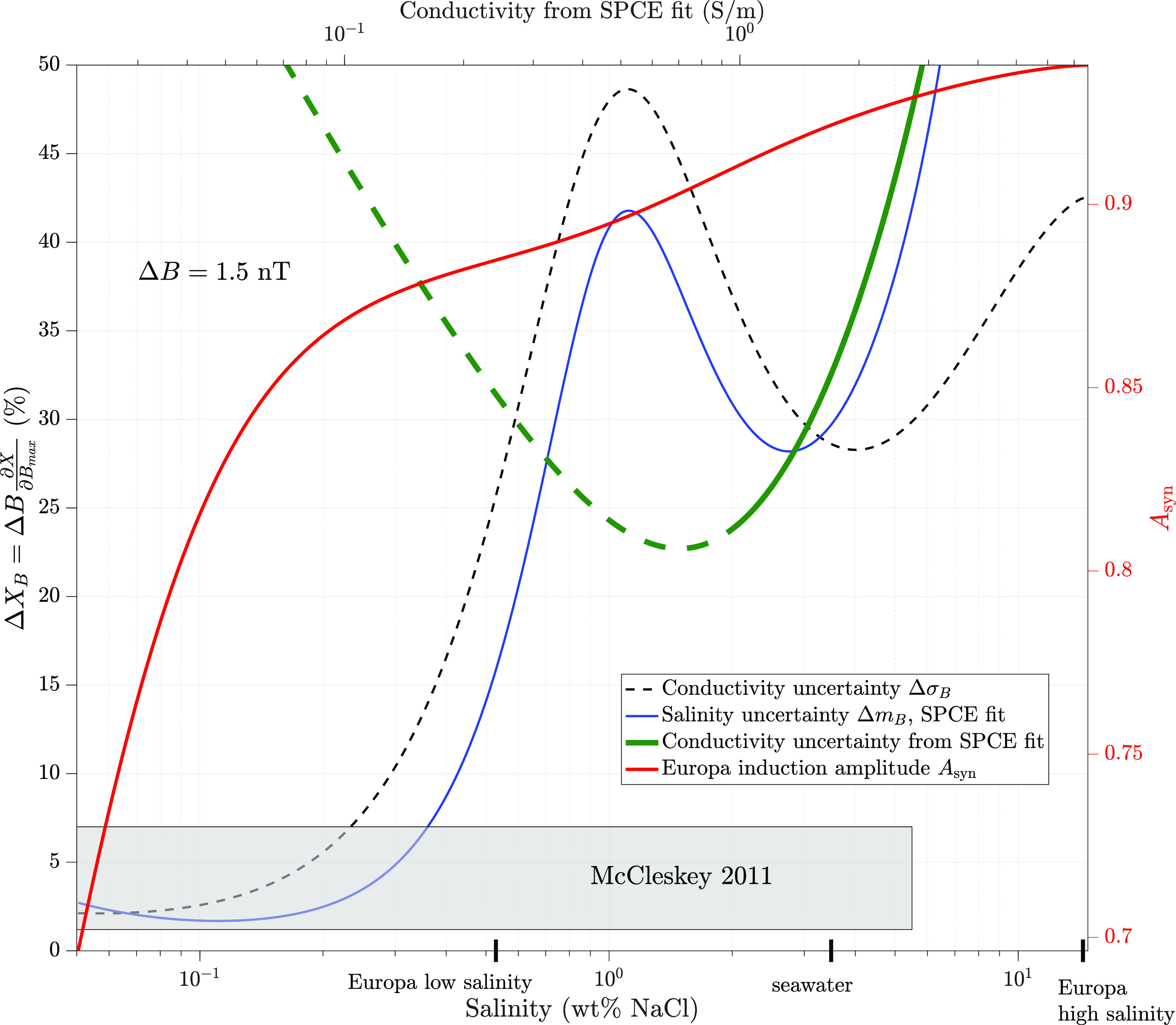
Comparison of uncertainties involved in mapping ocean conductivity to magnetic field measurements by spacecraft near Europa. Assuming an uncertainty of retrieval of 1.5 nT in the induced magnetic field vector components (Δ*B*), the conductivity of hypothesized ocean fluids must be predicted to an uncertainty Δσ_B_ at or below the line shown, or it will be a dominant source of uncertainty, greater than that of Europa Clipper’s magnetic induction investigation. A typical model of Europa is assumed^5^ for this calculation, with 20 km ice covering a uniform-conductivity ocean of thickness 150 km. The corresponding uncertainty in salinity Δ*m*_B_ is mapped from conductivity using our fit to SPC/E data at 269.33 K and 24.6 MPa, consistent with the conditions at the ice–ocean interface of a seawater ocean under 20 km of ice. Relative uncertainty in the conductivity evaluated from the SPC/E fit is also shown (thick green line) and demonstrates that our fit is not suitable for magnetic sounding investigations considering the values are over 20% in the entire fit range, dominant over much of the range. The solid portion of the curve is the region of the model data—the other data are extrapolated but are in basic agreement with available measurements, as noted above. For comparison, the red curve shows the induction amplitude for Europa (up to a maximum of 1), with black ticks along the salinity *x*-axis showing commonly regarded limits of salinity for its ocean. Gray box: the reported uncertainty range from the measured conductivities of laboratory solutions by McCleskey.^31^

Figure 6 shows the range of uncertainty in the conductivity given the ocean parameters inferred from magnetic data near Europa. Equivalent limiting uncertainties for both salinity (Δ*m*_B_, solid blue lines) and conductivity (Δσ_B_, black dashed lines) are plotted, based on an approximate mapping from NaCl salinity to conductivity using the fit from our SPC/E MD simulations. The conductivity of aqueous fluids must be predicted in forward models to a precision below the black dashed lines (≲ 2% at low concentrations) so as not to be a significant source of uncertainty in magnetic sounding investigations. Laboratory measurements and/or MD simulations therefore require precision that lies below these curves to best support such investigations. The salinity coverage and the uncertainties assessed for the relevant measurements by McCleskey^31^ (gray rectangle) are mostly adequate by this standard, neglecting that they do not account for the influence of pressure described here. We omit relevant measurements by Pan et al.^15^ from the plot, owing to open questions about the interpretation of the above data and because they do not include measurements below 140 MPa relevant to Europa’s ocean. We consider the predicted conductivities of the fit to our SPC/E results (Table 1) to have an unacceptable uncertainty for the analysis of the magnetic induction data.

Our calculations (Figure 6) demonstrate that interpreting *Europa Clipper* magnetic measurements will require electrical conductivity reference data that are accurate to within 2% in the conductivity range approaching the lower expected limit of 0.1 S/m.^4^ Uncertainties that exceed 10% may be adequate for ocean conductivities above about 1 S/m. Recent measurements in aqueous MgSO_4_ and NaCl by Pan et al.^40^ and Pan et al.,^15^ mostly near 1 S/m, may be suitable for interpreting magnetic induction results, but it must be emphasized that those data are not comprehensive across the fluid stability range, and empirical fits to the data presented by the authors do not include data along the full melting curves that remain unavailable to date at the required precision. In the future, the regression fits based on our SPC/E MD results from this work may be implemented into PlanetProfile along with a new NaCl equation of state to perform a self-consistent sensitivity analysis that incorporates Europa pressure conditions. The current sensitivity analysis provides a figure of merit for assessing the needed accuracy of measurements under temperature and pressure conditions consistent with published models of Europa.

Our regression fits based on SPC/E MD results provide a preliminary survey of conductivity under ocean world conditions over the broad range of possible conditions for one of the most likely constituent ionic systems. The observed trends can guide future laboratory measurements and provide a basis of comparison for follow-up computational studies. Comparing electrical conductivity and density values at ambient pressure with prior experimental studies provides useful insights into conductivity trends at high pressure, and the simulations show a strong temperature dependence of conductivity at all pressures. The decreasing conductivity observed with pressure is statistically significant across the entire range of pressures studied, 0–2000 MPa. Our simulations support the idea that conductivity varies most strongly with temperature and concentration compared to pressure—though variations with pressure can be large enough to influence the interpretation of magnetic data.^5^ Quantifying these dependencies is vital to the interpretation of magnetic signals.

Future experiments may be conducted under low-temperature and high-salinity conditions relevant to Europa’s ocean to achieve higher precision data in the pressure range of Europa’s ocean (0–200 MPa). Increased attention to theoretical studies of high-concentration aqueous systems in recent years^41,42^ may help to understand the conductivity behavior of high-salinity oceans. A comprehensive database of electrical conductivity experiments for a multitude of aqueous solutions, including high salinity and low temperature, as well as the dependence on pressure, will provide a framework from which to forward-model the induced field at icy moons and compare with signals measured by future spacecraft.

## Supporting Information Available

Files available: The Supporting Information is available free of charge at https://pubs.acs.org/doi/10.1021/acsearthspacechem.3c00345.

Tables of regression model results and the input water model properties for each MD simulation run needed to reproduce the presented results (PDF)
Code for the computation of the noise-equivalent increment of conductivity for Europa (ZIP)
