# Supplementary material for: Electrical Conductivity of Subsurface Ocean Analogue Solutions from Molecular Dynamics Simulations
Source: ACS Earth Space Chem. 2024 Jun 8;8(6):1146–53. doi: 10.1021/acsearthspacechem.3c00345 (PMC11194852; doi:10.1021/acsearthspacechem.3c00345)
Supplement: Supplementary file 1 — sp3c00345_si_001.pdf [file sp3c00345_si_001.pdf]

Supplemental Text for "Electrical conductivity of subsurface ocean analog solutions from molecular dynamics simulations"

Catherine A. Psarakis,[*,†,‡] Timothy Tizhe. Fidelis,[¶] Keith B. Chin,[‡]
Baptiste Journaux,[§] Abby Kavner,[†] Pranab Sarker,[∥] Marshall J. Styczinski,[⊥]
Steven D. Vance,[‡] and Tao Wei[∥]

†*University of California, Los Angeles, Los Angeles, CA 90095 USA*

‡*Jet Propulsion Laboratory, California Institute of Technology, Pasadena, CA 91011 USA*

¶*Howard University, Washington, DC 20059 USA*

§*University of Washington, Seattle, Seattle, WA 98195 USA*

∥*University of South Carolina, Columbia, SC 29208 USA*

⊥*Blue Marble Space Institute of Science, Seattle, Washington 98104 USA*

E-mail: catherine.psarakis@ucla.edu

Table S1: Regression model results of density (ρ) and electrical conductivity (σ) vs. T in K, P in MPa, and m in wt% NaCl for the respective simulations using the SPC/E and TIP4P water models. A linear regression was used for density, while for conductivity a custom regression (after Eq. 3) was used.

ρ : **SPC/E**	Slope	Std. Error	t value
Constant	1206.8150	39.880	30.261
T (K)	−0.5970	0.140	−4.265
P (MPa)	0.1872	0.005	39.748
wt% NaCl	8.6179	0.797	10.815
**Model Fit**			
R^2:	0.961		
ρ : **TIP4P**	Slope	Std. Error	t value
Constant	1208.3956	43.916	27.516
T (K)	−0.5871	0.154	−3.823
P (MPa)	0.1924	0.005	37.835
wt% NaCl	8.6349	0.817	10.572
**Model Fit**			
R^2:	0.957		

wt% **fit parameter**	**SPC/E fit**	**TIP4P fit**	**combined**
A_1	0.021 98	0.030 49	0.004 369
A_2	−4.974	−6.758	−0.2470
A_3	18.34	7.205	21.61
A_4	62.32	152.4	168.0
A_5	−0.049 60	−0.018 53	−0.020 38
n	1.257	1.064	1.865
R^2 value	0.9861	0.9699	0.8436
Std. error (S/m)	0.3723	0.7096	1.515

Table S2: Regression model results of electrical conductivity (σ) vs. T in K, P in MPa, and m in molal ($\mathrm{mol\,NaCl/kg\,H_2O}$) for the respective simulations using the SPC/E and TIP4P water models. We used the same equation for the regression (after Eq. 3) for these fits as in Table S1, but NaCl salinities are instead expressed in molal. Goodness-of-fit parameters are nearly identical for both salinity units.

molal **fit parameter**	**SPC/E fit**	**TIP4P fit**	**combined**
A_1	0.1844	0.1866	0.087 95
A_2	−41.72	−41.36	−0.028 33
A_3	96.22	39.74	123.9
A_4	63.91	149.7	165.9
A_5	−0.049 25	−0.018 88	−0.020 72
n	1.198	1.018	1.877
R^2 value	0.9862	0.9700	0.8447
Std. error (S/m)	0.3720	0.7080	1.510

Table S3: Covariance matrices for regression of fit to conductivity with m in wt%.

SPC/E only

$$\begin{bmatrix} 2.027\times10^{-5} & -4.900\times10^{-3} & -2.218\times10^{-2} & -1.945\times10^{-2} & -3.483\times10^{-6} & -7.567\times10^{-4} \\ -4.900\times10^{-3} & 1.192 & 4.551 & 1.291\times10^{1} & 2.572\times10^{-3} & 1.776\times10^{-1} \\ -2.218\times10^{-2} & 4.551 & 1.229\times10^{2} & -9.384\times10^{2} & -1.989\times10^{-1} & 1.489 \\ -1.945\times10^{-2} & 1.291\times10^{1} & -9.384\times10^{2} & 9.560\times10^{3} & 2.011 & -5.491 \\ -3.483\times10^{-6} & 2.572\times10^{-3} & -1.989\times10^{-1} & 2.011 & 4.296\times10^{-4} & -1.200\times10^{-3} \\ -7.567\times10^{-4} & 1.776\times10^{-1} & 1.489 & -5.491 & -1.200\times10^{-3} & 3.299\times10^{-2} \end{bmatrix}$$

TIP4P only

$$\begin{bmatrix} 6.573\times10^{-5} & -1.720\times10^{-2} & -4.213\times10^{-3} & -3.466\times10^{-1} & -4.493\times10^{-5} & -1.089\times10^{-3} \\ -1.720\times10^{-2} & 4.556 & -5.781\times10^{-1} & 1.126\times10^{2} & 1.460\times10^{-2} & 2.571\times10^{-1} \\ -4.213\times10^{-3} & -5.781\times10^{-1} & 6.244\times10^{1} & -7.512\times10^{2} & -9.817\times10^{-2} & 1.096 \\ -3.466\times10^{-1} & 1.126\times10^{2} & -7.512\times10^{2} & 1.165\times10^{4} & 1.513 & -6.899 \\ -4.493\times10^{-5} & 1.460\times10^{-2} & -9.817\times10^{-2} & 1.513 & 2.015\times10^{-4} & -9.225\times10^{-4} \\ -1.089\times10^{-3} & 2.571\times10^{-1} & 1.096 & -6.899 & -9.225\times10^{-4} & 3.527\times10^{-2} \end{bmatrix}$$

combined

$$\begin{bmatrix} 6.811\times10^{-5} & -1.691\times10^{-2} & -5.797\times10^{-2} & -3.633\times10^{-2} & -7.113\times10^{-6} & -3.525\times10^{-3} \\ -1.691\times10^{-2} & 4.264 & 1.219\times10^{1} & 1.537\times10^{1} & 2.813\times10^{-3} & 8.089\times10^{-1} \\ -5.797\times10^{-2} & 1.219\times10^{1} & 1.406\times10^{2} & -2.303\times10^{2} & -3.857\times10^{-2} & 5.524 \\ -3.633\times10^{-2} & 1.537\times10^{1} & -2.303\times10^{2} & 7.791\times10^{2} & 1.311\times10^{-1} & -5.286 \\ -7.113\times10^{-6} & 2.813\times10^{-3} & -3.857\times10^{-2} & 1.311\times10^{-1} & 3.333\times10^{-5} & -8.769\times10^{-4} \\ -3.525\times10^{-3} & 8.089\times10^{-1} & 5.524 & -5.286 & -8.769\times10^{-4} & 2.549\times10^{-1} \end{bmatrix}$$

Table S4: Covariance matrices for regression of fit to conductivity with m in molal.

**SPC/E only**					
5.651×10^{-4}	-1.338×10^{-1}	1.243	-1.654	-3.557×10^{-4}	3.138×10^{-3}
-1.338×10^{-1}	3.254×10^{1}	-3.025×10^{2}	4.462×10^{2}	9.556×10^{-2}	-6.579×10^{-1}
1.243	-3.025×10^{2}	2.944×10^{3}	-4.421×10^{3}	-9.360×10^{-1}	6.293
-1.654	4.462×10^{2}	-4.421×10^{3}	8.906×10^{3}	1.871	-3.945
-3.557×10^{-4}	9.556×10^{-2}	-9.360×10^{-1}	1.871	3.996×10^{-4}	-8.680×10^{-4}
3.138×10^{-3}	-6.579×10^{-1}	6.293	-3.945	-8.680×10^{-4}	2.726×10^{-2}
**TIP4P only**					
1.584×10^{-3}	-4.144×10^{-1}	1.566	-3.958	-5.214×10^{-4}	3.817×10^{-3}
-4.144×10^{-1}	1.115×10^{2}	-3.943×10^{2}	1.085×10^{3}	1.425×10^{-1}	-7.543×10^{-1}
1.566	-3.943×10^{2}	1.671×10^{3}	-3.787×10^{3}	-4.946×10^{-1}	5.176
-3.958	1.085×10^{3}	-3.787×10^{3}	1.108×10^{4}	1.438	-5.825
-5.214×10^{-4}	1.425×10^{-1}	-4.946×10^{-1}	1.438	1.917×10^{-4}	-7.809×10^{-4}
3.817×10^{-3}	-7.543×10^{-1}	5.176	-5.825	-7.809×10^{-4}	3.080×10^{-2}
**combined**					
1.801×10^{-2}	-6.097	-2.012	-1.697	-3.086×10^{-4}	-3.645×10^{-2}
-6.097	2.128×10^{3}	1.141×10^{3}	3.991×10^{2}	7.276×10^{-2}	1.521×10^{1}
-2.012	1.141×10^{3}	3.611×10^{3}	-1.133×10^{3}	-1.900×10^{-1}	2.520×10^{1}
-1.697	3.991×10^{2}	-1.133×10^{3}	6.922×10^{2}	1.167×10^{-1}	-4.664
-3.086×10^{-4}	7.276×10^{-2}	-1.900×10^{-1}	1.167×10^{-1}	3.123×10^{-5}	-7.732×10^{-4}
-3.645×10^{-2}	1.521×10^{1}	2.520×10^{1}	-4.664	-7.732×10^{-4}	2.096×10^{-1}

Table S5: Water model properties

Water Model	T (K)	P (MPa)	wt% NaCl	molality (mol/kg)	ion pairs	total # particles	Density (kg/m^3)	Conductivity (S/m)
SPC/E	273	0.1	2	0.35	62	9896	1027.79	1.94
SPC/E	273	100	2	0.35	62	9896	1067.87	2.02
SPC/E	273	200	2	0.35	62	9896	1102.69	2.17
SPC/E	263	200	2	0.35	62	9896	1106.51	1.74
SPC/E	252	200	2	0.35	62	9896	1110.26	1.09
SPC/E	273	0.1	10.64	2.04	338	9896	1108.35	7.55
SPC/E	266	0.1	10.64	2.04	338	9896	1111.79	6.01
SPC/E	273	100	10.64	2.04	338	9896	1142.37	7.26
SPC/E	263	100	10.64	2.04	338	9896	1147.01	5.50
SPC/E	257	100	10.64	2.04	338	9896	1149.54	4.46
SPC/E	273	200	10.64	2.04	338	9896	1171.72	7.49
SPC/E	263	200	10.64	2.04	338	9896	1176.35	5.48
SPC/E	252	200	10.64	2.04	338	9896	1181.08	3.76
SPC/E	246	200	10.64	2.04	338	9896	1183.59	3.02
SPC/E	273	300	10	1.9	317	9896	1192.74	7.47
SPC/E	263	300	10	1.9	317	9896	1197.33	5.69
SPC/E	255	300	10	1.9	317	9896	1200.86	4.26
SPC/E	248	300	10	1.9	317	9896	1203.91	3.05
SPC/E	273	400	10	1.9	317	9896	1216.22	6.85

Continued on next page

Table S5: Water model properties (Continued)

Water Model	T (K)	P (MPa)	wt% NaCl	molality (mol/kg)	ion pairs	total # particles	Density (kg/m^3)	Conductivity (S/m)
SPC/E	252	400	10	1.9	317	9896	1225.79	3.83
SPC/E	273	500	10	1.9	317	9896	1237.48	7.19
SPC/E	266	500	10	1.9	317	9896	1240.79	5.81
SPC/E	258	500	10	1.9	317	9896	1244.51	4.28
SPC/E	278	700	10	1.9	317	9896	1272.79	7.96
SPC/E	271	700	10	1.9	317	9896	1276.1	6.39
SPC/E	293	900	10	1.9	317	9896	1298.32	9.81
SPC/E	285	900	10	1.9	317	9896	1302.16	8.42
SPC/E	298	1000	10	1.9	317	9896	1310.92	10.71
SPC/E	291	1000	10	1.9	317	9896	1314.24	9.22
SPC/E	307	1300	10	1.9	317	9896	1347.15	10.63
SPC/E	285	0.1	10	1.9	317	9896	1096.13	10.17
SPC/E	285	100	10	1.9	317	9896	1130.87	10.31
SPC/E	285	200	10	1.9	317	9896	1160.7	10.08
SPC/E	285	300	10	1.9	317	9896	1186.91	9.41
SPC/E	285	400	10	1.9	317	9896	1210.44	9.09
SPC/E	285	500	10	1.9	317	9896	1231.74	9.04
SPC/E	285	700	10	1.9	317	9896	1269.43	8.76
SPC/E	285	900	10	1.9	317	9896	1269.43	8.66

4

Continued on next page

Table S5: Water model properties (Continued)

Water Model	T (K)	P (MPa)	wt% NaCl	molality (mol/kg)	ion pairs	total # particles	Density (kg/m^3)	Conductivity (S/m)
SPC/E	298	0.1	5.15	0.93	160	9896	1044.68	7.52
SPC/E	298	100	5.15	0.93	160	9896	1082.99	8.12
SPC/E	298	200	5.15	0.93	160	9896	1115.61	8.04
SPC/E	298	300	5.15	0.93	160	9896	1144.13	7.94
SPC/E	298	400	5.15	0.93	160	9896	1169.43	8.12
SPC/E	298	500	5.15	0.93	160	9896	1192.23	7.40
SPC/E	298	700	5.15	0.93	160	9896	1232.12	7.09
SPC/E	298	900	5.15	0.93	160	9896	1266.38	7.41
SPC/E	298	1000	5.15	0.93	160	9896	1281.88	6.79
SPC/E	298	1300	5.15	0.93	160	9896	1323.46	6.41
SPC/E	298	1700	5.15	0.93	160	9896	1370.4	5.52
SPC/E	298	2000	5.15	0.93	160	9896	1401.02	5.03
SPC/E	298	0.1	10.64	2.04	338	9896	1094.16	13.57
SPC/E	298	100	10.64	2.04	338	9896	1129.11	13.09
SPC/E	298	200	10.64	2.04	338	9896	1158.92	13.62
SPC/E	298	300	10.64	2.04	338	9896	1185.07	12.86
SPC/E	298	400	10.64	2.04	338	9896	208.46	13.08
SPC/E	298	700	10.64	2.04	338	9896	1267.21	11.84
SPC/E	298	900	10.64	2.04	338	9896	1299.82	11.61

Continued on next page

Table S5: Water model properties (Continued)

Water Model	T (K)	P (MPa)	wt% NaCl	molality (mol/kg)	ion pairs	total # particles	Density (kg/m^3)	Conductivity (S/m)
SPC/E	298	1000	10.64	2.04	338	9896	1314.71	10.71
SPC/E	298	1300	10.64	2.04	338	9896	1355.03	9.80
SPC/E	298	1700	10.64	2.04	338	9896	1401.12	8.81
SPC/E	298	2000	10.64	2.04	338	9896	1431.43	8.77
TIP4P	298	0.1	5	0.9	157	10054	1042.5	9.54
TIP4P	298	100	5	0.9	157	10054	1084	9.45
TIP4P	298	200	5	0.9	157	10054	1118.36	9.44
TIP4P	298	300	5	0.9	157	10054	1147.9	9.45
TIP4P	298	400	5	0.9	157	10054	1173.88	9.77
TIP4P	298	500	5	0.9	157	10054	1197.16	9.69
TIP4P	298	900	5	0.9	157	10054	1272.38	9.67
TIP4P	298	1000	5	0.9	157	10054	1288.09	8.89
TIP4P	298	1300	5	0.9	157	10054	1330.2	9.14
TIP4P	298	1700	5	0.9	157	10054	1377.74	7.44
TIP4P	298	2000	5	0.9	157	10054	1408.76	6.78
TIP4P	298	0.1	10	1.9	321	10054	1091.18	16.04
TIP4P	298	100	10	1.9	321	10054	1128.76	15.79
TIP4P	298	200	10	1.9	321	10054	1160.12	16.38
TIP4P	298	300	10	1.9	321	10054	1187.24	16.33

Continued on next page

6

Table S5: Water model properties (Continued)

Water Model	T (K)	P (MPa)	wt% NaCl	molality (mol/kg)	ion pairs	total # particles	Density (kg/m^3)	Conductivity (S/m)
TIP4P	298	500	10	1.9	321	10054	1233.08	16.82
TIP4P	298	700	10	1.9	321	10054	1271.48	15.69
TIP4P	298	900	10	1.9	321	10054	1304.62	15.45
TIP4P	298	1000	10	1.9	321	10054	1319.78	14.63
TIP4P	298	1300	10	1.9	321	10054	1360.75	13.47
TIP4P	298	1700	10	1.9	321	10054	1407.53	12.64
TIP4P	298	2000	10	1.9	321	10054	1438.29	10.00
TIP4P	298	500	2.23	0.39	70	10054	1178.13	5.45
TIP4P	298	500	2.23	0.39	70	10054	1178.11	4.98
TIP4P	298	1000	2.23	0.39	70	10054	1271.39	4.99
TIP4P	273	0.1	2.23	0.39	70	10054	1027.73	2.97
TIP4P	273	100	2.23	0.39	70	10054	1071.58	2.99
TIP4P	263	100	2.23	0.39	70	10054	1075.09	2.24
TIP4P	263	200	2.23	0.39	70	10054	1112.51	2.61
TIP4P	252	200	2.23	0.39	70	10054	1116.57	1.61
TIP4P	273	0.1	10	1.9	321	10054	1106.48	9.95
TIP4P	266	0.1	10	1.9	321	10054	1109.9	7.00
TIP4P	273	100	10	1.9	321	10054	1142.75	9.38
TIP4P	263	100	10	1.9	321	10054	1147.5	7.63

7 Continued on next page

Table S5: Water model properties (Continued)

Water Model	T (K)	P (MPa)	wt% NaCl	molality (mol/kg)	ion pairs	total # particles	Density (kg/m^3)	Conductivity (S/m)
TIP4P	257	100	10	1.9	321	10054	1150.08	5.85
TIP4P	273	200	10	1.9	321	10054	1173.49	9.64
TIP4P	263	200	10	1.9	321	10054	1178.2	7.51
TIP4P	252	200	10	1.9	321	10054	1182.95	5.74
TIP4P	246	200	10	1.9	321	10054	1185.51	4.54
TIP4P	273	300	10	1.9	321	10054	1196.72	8.97
TIP4P	263	300	10	1.9	321	10054	1201.24	6.87
TIP4P	255	300	10	1.9	321	10054	1204.74	5.08
TIP4P	248	300	10	1.9	321	10054	1207.68	4.00
TIP4P	273	400	10	1.9	321	10054	1220.61	8.31
TIP4P	263	400	10	1.9	321	10054	1225.25	6.35
TIP4P	252	400	10	1.9	321	10054	1230.33	4.72
TIP4P	273	500	10	1.9	321	10054	1242.96	7.91
TIP4P	266	500	10	1.9	321	10054	1246.14	6.54
TIP4P	258	500	10	1.9	321	10054	1249.73	4.89
TIP4P	278	700	10	1.9	321	10054	1279.41	8.42
TIP4P	271	700	10	1.9	321	10054	1283.14	6.79
TIP4P	293	900	10	1.9	321	10054	1305.28	10.15
TIP4P	285	900	10	1.9	321	10054	1309.24	9.00

8 Continued on next page

Table S5: Water model properties (Continued)

Water Model	T (K)	P (MPa)	wt% NaCl	molality (mol/kg)	ion pairs	total # particles	Density (kg/m^3)	Conductivity (S/m)
TIP4P	298	1000	10	1.9	321	10054	1317.85	11.79
TIP4P	291	1000	10	1.9	321	10054	1321.49	10.04
TIP4P	307	1300	10	1.9	321	10054	1352.67	11.78
TIP4P	285	0.1	10	1.9	321	10054	1095.05	10.82
TIP4P	285	100	10	1.9	321	10054	1130.42	10.69
TIP4P	285	200	10	1.9	321	10054	1160.79	10.35
TIP4P	285	300	10	1.9	321	10054	1187.87	9.97
TIP4P	285	400	10	1.9	321	10054	1211.29	10.06
TIP4P	285	500	10	1.9	321	10054	1232.46	9.48
TIP4P	285	700	10	1.9	321	10054	1269.8	9.37
TIP4P	285	900	10	1.9	321	10054	1302.38	9.16
TIP4P	298	0.1	5.15	0.93	160	10054	1044.72	7.48
TIP4P	298	100	5.15	0.93	160	10054	1083.31	7.63
TIP4P	298	200	5.15	0.93	160	10054	1115.4	7.83
TIP4P	298	300	5.15	0.93	160	10054	1143.74	7.86
TIP4P	298	400	5.15	0.93	160	10054	1168.66	7.92
TIP4P	298	500	5.15	0.93	160	10054	1190.43	7.78
TIP4P	298	700	5.15	0.93	160	10054	1230.66	7.64
TIP4P	298	900	5.15	0.93	160	10054	1265.27	7.63

9 Continued on next page

Table S5: Water model properties (Continued)

Water Model	T (K)	P (MPa)	wt% NaCl	molality (mol/kg)	ion pairs	total # particles	Density (kg/m^3)	Conductivity (S/m)
TIP4P	298	1000	5.15	0.93	160	10054	1280.45	7.21
TIP4P	298	1300	5.15	0.93	160	10054	1321.18	7.16
TIP4P	298	1700	5.15	0.93	160	10054	1368.41	6.15
TIP4P	298	2000	5.15	0.93	160	10054	1399.42	5.64
TIP4P	298	0.1	10.64	2.04	338	10054	1093.22	13.84
TIP4P	298	100	10.64	2.04	338	10054	1128.19	14.11
TIP4P	298	200	10.64	2.04	338	10054	1158.01	14.05
TIP4P	298	300	10.64	2.04	338	10054	1184.18	13.90
TIP4P	298	400	10.64	2.04	338	10054	1208.05	14.05
TIP4P	298	500	10.64	2.04	338	10054	1230.41	13.58
TIP4P	298	700	10.64	2.04	338	10054	1267.59	12.68
TIP4P	298	900	10.64	2.04	338	10054	1300.09	12.55
TIP4P	298	1000	10.64	2.04	338	10054	1314.94	11.74
TIP4P	298	1300	10.64	2.04	338	10054	1355.51	10.88
TIP4P	298	1700	10.64	2.04	338	10054	1401.85	9.58
TIP4P	298	2000	10.64	2.04	338	10054	1432.82	9.13
TIP4P	298	500	2.23	0.39	70	10054	1180.97	5.74
TIP4P	298	1000	2.23	0.39	70	10054	1273.46	5.71
TIP4P	273	0.1	2.23	0.39	70	10054	1028.23	2.97

Continued on next page

Table S5: Water model properties (Continued)

Water Model	T (K)	P (MPa)	wt% NaCl	molality (mol/kg)	ion pairs	total # particles	Density (kg/m^3)	Conductivity (S/m)
TIP4P	273	100	2.23	0.39	70	10054	1071.63	2.99
TIP4P	263	100	2.23	0.39	70	10054	1075.27	2.25
TIP4P	263	200	2.23	0.39	70	10054	1112.96	2.62
TIP4P	252	200	2.23	0.39	70	10054	1116.85	1.61
TIP4P	273	0.1	10	1.9	321	10054	1106.84	9.94
TIP4P	266	0.1	10	1.9	321	10054	1110.26	7.00
TIP4P	273	100	10	1.9	321	10054	1143.29	9.36
TIP4P	263	100	10	1.9	321	10054	1147.93	7.61
TIP4P	257	100	10	1.9	321	10054	1150.54	5.84
TIP4P	273	200	10	1.9	321	10054	1173.88	9.64
TIP4P	263	200	10	1.9	321	10054	1178.63	7.51
TIP4P	252	200	10	1.9	321	10054	1183.39	5.74
TIP4P	273	300	10	1.9	321	10054	1196.92	8.97
TIP4P	263	300	10	1.9	321	10054	1201.64	6.88
TIP4P	255	300	10	1.9	321	10054	1205.1	5.08
TIP4P	248	300	10	1.9	321	10054	1207.9	4.00
TIP4P	273	400	10	1.9	321	10054	1221.08	8.29
TIP4P	263	400	10	1.9	321	10054	1225.73	6.35
TIP4P	252	400	10	1.9	321	10054	1230.82	4.72

Continued on next page

Table S5: Water model properties (Continued)

Water Model	T (K)	P (MPa)	wt% NaCl	molality (mol/kg)	ion pairs	total # particles	Density (kg/m^3)	Conductivity (S/m)
TIP4P	273	500	10	1.9	321	10054	1243.48	7.91
TIP4P	266	500	10	1.9	321	10054	1246.68	6.54
TIP4P	258	500	10	1.9	321	10054	1250.25	4.89
TIP4P	278	700	10	1.9	321	10054	1279.99	8.42
TIP4P	271	700	10	1.9	321	10054	1283.74	6.79
TIP4P	293	900	10	1.9	321	10054	1305.94	10.16
TIP4P	285	900	10	1.9	321	10054	1309.92	9.00
TIP4P	298	1000	10	1.9	321	10054	1318.63	11.80
TIP4P	291	1000	10	1.9	321	10054	1322.29	10.05
TIP4P	307	1300	10	1.9	321	10054	1353.51	11.79
TIP4P	285	0.1	10	1.9	321	10054	1095.39	10.82
TIP4P	285	100	10	1.9	321	10054	1130.85	10.69
TIP4P	285	200	10	1.9	321	10054	1161.19	10.36
TIP4P	285	300	10	1.9	321	10054	1188.24	9.97
TIP4P	285	400	10	1.9	321	10054	1211.64	10.06
TIP4P	285	500	10	1.9	321	10054	1232.83	9.48
TIP4P	285	700	10	1.9	321	10054	1270.17	9.37
TIP4P	285	900	10	1.9	321	10054	1302.76	9.16
TIP4P	298	0.1	5.15	0.93	160	10054	1045.21	7.48

Continued on next page

Table S5: Water model properties (Continued)

Water Model	T (K)	P (MPa)	wt% NaCl	molality (mol/kg)	ion pairs	total # particles	Density (kg/m^3)	Conductivity (S/m)
TIP4P	298	100	5.15	0.93	160	10054	1083.85	7.63
TIP4P	298	200	5.15	0.93	160	10054	1116.06	7.84
TIP4P	298	300	5.15	0.93	160	10054	1144.48	7.87
TIP4P	298	400	5.15	0.93	160	10054	1169.44	7.93
TIP4P	298	500	5.15	0.93	160	10054	1191.24	7.79
TIP4P	298	700	5.15	0.93	160	10054	1231.58	7.65
TIP4P	298	900	5.15	0.93	160	10054	1266.25	7.63
TIP4P	298	1000	5.15	0.93	160	10054	1281.42	7.22
TIP4P	298	1300	5.15	0.93	160	10054	1322.16	7.17
TIP4P	298	1700	5.15	0.93	160	10054	1369.41	6.15
TIP4P	298	2000	5.15	0.93	160	10054	1400.42	5.65
TIP4P	298	0.1	10.64	2.04	338	10054	1093.72	13.83
TIP4P	298	100	10.64	2.04	338	10054	1128.7	14.11
TIP4P	298	200	10.64	2.04	338	10054	1158.53	14.05
TIP4P	298	300	10.64	2.04	338	10054	1184.69	13.90
TIP4P	298	400	10.64	2.04	338	10054	1208.57	14.05
TIP4P	298	500	10.64	2.04	338	10054	1230.94	13.58
TIP4P	298	700	10.64	2.04	338	10054	1268.12	12.68
TIP4P	298	900	10.64	2.04	338	10054	1300.63	12.55

13 Continued on next page

Table S5: Water model properties (Continued)

Water Model	T (K)	P (MPa)	wt% NaCl	molality (mol/kg)	ion pairs	total # particles	Density (kg/m^3)	Conductivity (S/m)
TIP4P	298	1000	10.64	2.04	338	10054	1315.48	11.74
TIP4P	298	1300	10.64	2.04	338	10054	1356.05	10.88
TIP4P	298	1700	10.64	2.04	338	10054	1402.39	9.58
TIP4P	298	2000	10.64	2.04	338	10054	1433.36	9.13
TIP4P	298	500	2.23	0.39	70	10054	1181.32	5.74
TIP4P	298	1000	2.23	0.39	70	10054	1273.81	5.71
TIP4P	273	0.1	2.23	0.39	70	10054	1028.57	2.97
TIP4P	273	100	2.23	0.39	70	10054	1072.01	2.99
TIP4P	263	100	2.23	0.39	70	10054	1075.66	2.25
TIP4P	263	200	2.23	0.39	70	10054	1113.35	2.62
TIP4P	252	200	2.23	0.39	70	10054	1117.24	1.61
TIP4P	273	0.1	10	1.9	321	10054	1107.23	9.94
TIP4P	266	0.1	10	1.9	321	10054	1110.65	7.00
TIP4P	273	100	10	1.9	321	10054	1143.68	9.36
TIP4P	263	100	10	1.9	321	10054	1148.32	7.61
TIP4P	257	100	10	1.9	321	10054	1150.93	5.84
TIP4P	273	200	10	1.9	321	10054	1174.28	9.64
TIP4P	263	200	10	1.9	321	10054	1179.03	7.51
TIP4P	252	200	10	1.9	321	10054	1183.79	5.74

14

Continued on next page

Table S5: Water model properties (Continued)

Water Model	T (K)	P (MPa)	wt% NaCl	molality (mol/kg)	ion pairs	total # particles	Density (kg/m^3)	Conductivity (S/m)
TIP4P	273	300	10	1.9	321	10054	1197.32	8.97
TIP4P	263	300	10	1.9	321	10054	1202.04	6.88
TIP4P	255	300	10	1.9	321	10054	1205.5	5.08
TIP4P	248	300	10	1.9	321	10054	1208.3	4.00
TIP4P	273	400	10	1.9	321	10054	1221.48	8.29
TIP4P	263	400	10	1.9	321	10054	1226.13	6.35
TIP4P	252	400	10	1.9	321	10054	1231.21	4.72
TIP4P	273	500	10	1.9	321	10054	1243.84	7.91
TIP4P	266	500	10	1.9	321	10054	1247.03	6.54
TIP4P	258	500	10	1.9	321	10054	1250.62	4.89
TIP4P	278	700	10	1.9	321	10054	1280.33	8.42
TIP4P	271	700	10	1.9	321	10054	1284.06	6.79
TIP4P	293	900	10	1.9	321	10054	1306.2	10.15
TIP4P	285	900	10	1.9	321	10054	1310.17	9.00
TIP4P	298	1000	10	1.9	321	10054	1318.78	11.79
TIP4P	291	1000	10	1.9	321	10054	1322.42	10.04
TIP4P	307	1300	10	1.9	321	10054	1353.64	11.79
TIP4P	285	0.1	10	1.9	321	10054	1095.6	10.82
TIP4P	285	100	10	1.9	321	10054	1131.07	10.69

Continued on next page

Table S5: Water model properties (Continued)

Water Model	T (K)	P (MPa)	wt% NaCl	molality (mol/kg)	ion pairs	total # particles	Density (kg/m^3)	Conductivity (S/m)
TIP4P	285	200	10	1.9	321	10054	1161.41	10.36
TIP4P	285	300	10	1.9	321	10054	1188.46	9.97
TIP4P	285	400	10	1.9	321	10054	1211.85	10.06
TIP4P	285	500	10	1.9	321	10054	1233.04	9.48
TIP4P	285	700	10	1.9	321	10054	1270.38	9.37
TIP4P	285	900	10	1.9	321	10054	1302.97	9.16

Table S6: Conditions for conductivity data from the literature corresponding to Figure 5.

Method	P (MPa)	T (K)	wt% NaCl
SPC/E MD Sims from this work	0.1	298	5.15
SPC/E MD Sims from this work	100	298	5.15
SPC/E MD Sims from this work	200	298	5.15
SPC/E MD Sims from this work	300	298	5.15
SPC/E MD Sims from this work	400	298	5.15
SPC/E MD Sims from this work	500	298	5.15
SPC/E MD Sims from this work	700	298	5.15
SPC/E MD Sims from this work	900	298	5.15
SPC/E MD Sims from this work	1000	298	5.15
SPC/E MD Sims from this work	1300	298	5.15
SPC/E MD Sims from this work	1700	298	5.15
SPC/E MD Sims from this work	2000	298	5.15
Simnyo and Keppler (2016) Experiments	0	300	5.36
Simnyo and Keppler (2016) Experiments	26	300	5.36
Simnyo and Keppler (2016) Experiments	48	300	5.15
McCleskey et al (2011) Experiments	0.1	298	5.52
Guo and Keppler (2019) Experiments	1000	298	4.41
Guo and Keppler (2019) Experiments	2000	298	4.2
Adams and Hall (1930) Experiments	400	298	5.08

Continued on next page

Table S6: Conditions for conductivity data from the literature corresponding to Figure 5. (Continued)

**Method**	**P (MPa)**	**T (K)**	**wt% NaCl**
Pan et al (2021) SPC/E MD Sims	0	295	5
Pan et al (2021) SPC/E MD Sims	100	295	5
Pan et al (2021) SPC/E MD Sims	200	295	5
Pan et al (2021) SPC/E MD Sims	520	295	5
Defense Lab (1964) Experiments	0.1	298	5
